# Prevalence, incidence and carrier frequency of 5q–linked spinal muscular atrophy – a literature review

**DOI:** 10.1186/s13023-017-0671-8

**Published:** 2017-07-04

**Authors:** Ingrid E. C. Verhaart, Agata Robertson, Ian J. Wilson, Annemieke Aartsma-Rus, Shona Cameron, Cynthia C. Jones, Suzanne F. Cook, Hanns Lochmüller

**Affiliations:** 1 John Walton Muscular Dystrophy Research Centre, Institute of Genetic Medicine, Newcastle University, Newcastle upon Tyne, UK; 2 Institute of Genetic Medicine, Newcastle University, Newcastle upon Tyne, UK; 3 Biogen MA Inc., Cambridge, USA; 4 Epidemiology Associates LLC, Chapel Hill, USA; 5 John Walton Muscular Dystrophy Research Centre, MRC Centre for Neuromuscular Diseases, Institute of Genetic Medicine, Newcastle University, Central Parkway, Newcastle upon Tyne, NE1 3BZ UK

**Keywords:** Spinal muscular atrophy, Prevalence, Incidence, Carrier frequency, Ethnic background

## Abstract

Spinal muscular atrophy linked to chromosome 5q (SMA) is a recessive, progressive, neuromuscular disorder caused by bi-allelic mutations in the *SMN1* gene, resulting in motor neuron degeneration and variable presentation in relation to onset and severity. A prevalence of approximately 1–2 per 100,000 persons and incidence around 1 in 10,000 live births have been estimated with SMA type I accounting for around 60% of all cases. Since SMA is a relatively rare condition, studies of its prevalence and incidence are challenging. Most published studies are outdated and therefore rely on clinical rather than genetic diagnosis. Furthermore they are performed in small cohorts in small geographical regions and only study European populations. In addition, the heterogeneity of the condition can lead to delays and difficulties in diagnosing the condition, especially outside of specialist clinics, and contributes to the challenges in understanding the epidemiology of the disease. The frequency of unaffected, heterozygous carriers of the *SMN1* mutations appears to be higher among Caucasian and Asian populations compared to the Black (Sub-Saharan African ancestry) population. However, carrier frequencies cannot directly be translated into incidence and prevalence, as very severe (death in utero) and very mild (symptom free in adults) phenotypes carrying bi-allelic *SMN1* mutations exist, and their frequency is unknown.

More robust epidemiological data on SMA covering larger populations based on accurate genetic diagnosis or newborn screening would be helpful to support planning of clinical studies, provision of care and therapies and evaluation of outcomes.

**Electronic supplementary material:**

The online version of this article (doi:10.1186/s13023-017-0671-8) contains supplementary material, which is available to authorized users.

## Background

Spinal muscular atrophy (SMA) is characterised by degeneration of the alpha motor neurons of the spinal cord anterior horn cells, leading to progressive proximal muscle weakness and atrophy and, in the most severe types, paralysis.

The clinical phenotype of SMA is heterogeneous, ranging from a severe to a mild phenotype. It is generally divided into three main subtypes: type I (also called Werdnig Hoffmann disease), type II and, type III (also called Kugelberg Welander disease). However, these phenotypes are seen more as a continuum rather than as distinct subtypes and sometimes further subtypes at both ends of the spectrum are observed. SMA type 0 is a very severe form with onset in utero, reduced or absent movements, contractures, and requirement for mechanical ventilation support at birth and death before six months of age, while SMA type IV is a mild late (adult) onset form that has a normal life expectancy [1, 2]. An overview of the different subtypes is given in Table 1.

**Table 1:** Clinical classification of spinal muscular atrophy

SMA type	OMIM number	Age of onset	Highest achieved motor function	Natural age of death
0	-	Prenatal/ Foetal	Nil	<6 months
I	253300	<6 months	Sit with support only	<2 years
II	253550	6–18 months	Sit independently	>2 years
III	253400	>18 months	Stand and walk	Adulthood
IV	271150	Adult (2nd or 3rd decade)	Walk during adulthood	Adult

*OMIM* Online Mendelian Inheritance in Man

SMA is inherited in an autosomal recessive manner. In most cases it is caused by mutations in the survival motor neuron 1 (*SMN1, SMN* ^*T*^, telomeric) gene, located on chromosome 5q13.2 [3]. In rare cases (~4%) SMA is caused by mutation in another gene (non-5q SMA). The majority of the patients (92%) have a homozygous deletion of *SMN1*. In the remaining patients small mutations that abolish the production of the SMN protein are found, mostly in a combination with an *SMN1* deletion (~4%) [4, 5]. A centromeric homologue of the gene, *SMN2*, (previously also called *SMN* ^*C*^ or ^*C*^ *BCD541*) is present in humans. *SMN2* differs from *SMN1* by five nucleotides of which only one (an 840C➔T transition at exon 6–7) lies in the coding sequence and is transitionally silent. This change and a change in intron 7 cause exon 7 of the *SMN2* transcript to be poorly recognized by the splicing machinery, resulting in the skipping of this exon in the majority of transcripts. This results in a frame-shift and production of a protein with a different C-terminal end, which is unstable and non-functional [3, 6]. Since exon 7 is sometimes included in *SMN2* transcripts, some full-length SMN protein can be produced, albeit as very low levels (~10–20%) that are insufficient to prevent disease. The number of *SMN2* copies varies within the general population, and is inversely associated with disease severity as having more *SMN2* copies ensures that the absolute amount of SMN protein that is produced is higher. Notably, *SMN2* defects in isolation do not seem to cause the disease [7–9]. Other modifiers that might play a role are *NAIP*, *H4F5*, *GTF2H2* and *PLS3* [10–15]. *NAIP*, *H4F5* and *GTF2H2* are thought to be a modifiers due to their proximity to the *SMN1* gene and *NAIP* also shows homology to apoptosis inhibitory proteins [12, 14, 16]. *PLS3* restores the function of the neuromuscular junction, by stabilizing F-actin-dependent endocytosis [17].

The first therapy for SMA, Spinraza (IONIS-SMNRx, nusinersen), has recently been approved by the Food and Drug Administration (FDA) in the US [18] and by the European Medicines Agency (EMA) in Europe [19]. Clinical trials for other potential therapies are progressing. As such, the knowledge about the frequency of the disease becomes even more important. This review provides an overview of what is currently known about the prevalence, incidence and carrier frequency of SMA.

## Methods

Published literature on prevalence, incidence or carrier frequency of SMA was identified through PubMed searches. Search terms were ‘spinal muscular atrophy’ OR ‘Werdnig Hoffmann’ OR ‘Kugelberg Welander’ AND ‘prevalence’ OR ‘incidence’, OR ‘carrier frequency’. No restrictions for language were used; however articles in other languages than English may be missed, due to the use of English search terms. Retrieved literature was scanned and all available articles performing a prevalence, incidence or carrier frequency study were used for this review. Additional publications were identified from references in the articles. Available literature published through 6th December 2016 was taken into account; no start date was used. For prevalence and incidence studies, all studies had determining the prevalence and/or incidence as primary goal. For carrier frequency studies also studies in which carrier frequency was determined for other purposes were included. All articles were appraised critically for accurate use of terminology and were reassigned if needed. For detailed methods on the analysis of carrier frequency differences between ethnic groups see Additional file 1.

## Prevalence and incidence of SMA

To date, only a few studies have been performed to assess the prevalence and incidence of SMA. Most of these have been conducted before 1995, when the disease causing gene was identified, therefore using clinical rather than genetic diagnosis as an inclusion criterion. Generally, an estimation of the incidence of all types of SMA of around 10 in 100,000 (1 in 10,000) live births is cited [20, 21].

### Prevalence

Prevalence is the number of living individuals with a disease at a given time. An overview of the studies examining the prevalence of SMA is provided in Table 2.

**Table 2:** Overview prevalence of SMA by subtype

Country	Time point	SMA			Type I			Quality and comparability				Reference
		No of patients	Population	per 100,000	No of patients	Population	per 100,000					
Norway (Southern + Eastern)	01/01/1983	24	573,762	4.18	1	573,762	0.17	Only included patients below 18 years of age				Tangsrud_1988 [25]
Sweden (Western)	31/12/2006	11	340,179	3.23	-	-	-	Genetic diagnosis used Only included patients below 16 years of age				Arkblad_2009 [23]
Sweden (Western)	01/01/1995	10	359,676	2.78	1	359,676	0.28	Only included patients below 16 years of age				Darin_2000 [24]
Estonia	31/12/2003	-	-	-	2	1,351,069	0.15	Genetic diagnosis used				Vaidla_2006 [34]
United Kingdom (Northern Ireland)	30/06/1994	22	1,573,282	1.40	-	-	-					Hughes_1996 [110]
United Kingdom (Northern England)	01/08/2007	56	2,991,517	1.87	3	2,991,517	0.10	Genetic diagnosis used in most cases				Norwood_2009 [32]
Germany (West‑Thüringen)	31/12/1987	-	-	-	3	1,778,200	0.17					Thieme_1993 [33]
Italy (Bologna)	31/12/1989	10	152,529	6.56	-	-	-	Only included patients below 20 years of age				Merlini_1992 [22]
Saudi Arabia (Northeast, Thugbah)	02/08/1989	3	22,630	13.26	1	22,630	4.42	In half of the cases parental consanguinity was observed				al Rajeh_1993 [29]
China (Hongkong)	30/06/2001	25	1,335,469	1.87	2	1,335,469	0.15	Partly used genetic diagnosis				Chung_2003 [31]
Canada (Toronto)	1962–1964	20.33	2,748,500	0.74	-	-	-	Average of a three-year period				Winsor_1971 [111]
Country	Time point	Type II			Type III			Type II + III			Quality and comparability	Reference
		No of patients	Population	per 100,000	No of patients	Population	per 100,000	No of patients	Population	per 100,000		
Norway (Southern + Eastern)	01/01/1983	21	573,762	3.66	2	573,762	0.35	23	573,762	4.01	Only included patients below 18 years of age	Tangsrud_1988 [25]
Sweden (Western)	01/01/1995	4	359,676	1.11	5	359,676	1.39	9	359,676	2.50	Only included patients below 16 years of age	Darin_2000 [24]
United Kingdom (Northern England)	01/08/2007	17	2,991,517	0.57	36	2,991,517	1.20	53	2,991,517	1.77	Genetic diagnosis used in most cases^a^	Norwood_2009 [32]
United Kingdom (Northeast England)	June 1971	-	-	-	-	-	-	30	2,488,810	1.21	Type II/III patients defined as: onset of disease between birth and 8 years of age, survival above 18 months of age	Pearn_1978 [41]
Germany (West‑Thüringen)	31/12/1980	-	-	-	-	-	-	29	1,785,239	1.62	Type II/III patients defined as: survival above 4 years of age Exact number of patients not mentioned^b^	Thieme_1994 [43]
Poland (Warsaw)	31/12/1985	-	-	-	-	-	-	21	1,659,385	1.26	Exact number of patients not mentioned^b^	Spiegler_1990 [42]
Libya (Benghazi)	31/12/1986	-	-	-	-	-	-	12	-	2.30	In half of the cases parental consanguinity was observed	Radhakrishan_1988 [48]
Saudi Arabia (Northeast, Thugbah)	02/08/1989	2	22,630	8.84	-	-	-	-	-	-	In half of the cases parental consanguinity was observed	al Rajeh_1993 [29]
China (Hongkong)	30/06/2001	9	1,335,469	0.67	14	1,335,469	1.05	23	1,335,469	1.72	Partly used genetic diagnosis	Chung_2003 [31]

Prevalence per 100,000 persons of the total population. Studies have been grouped by geographical region. Studies are based on clinical diagnosis, unless otherwise indicated. Indicated if described in article that different classification criteria than those in Table 1 were used

^a)^ 17 patients are classified as type III on clinical and neurophysiological findings in the absence of a confirmed mutation in the *SMN1* gene

^b)^ Number of patients not described in article, calculated based on prevalence and population number

When examining all types of SMA together, in most cases a prevalence of around 1–2 per 100,000 persons is observed. In some studies a somewhat higher prevalence was observed. A study from Bologna, Italy, in 1992 calculated a prevalence of 6.56 per 100,000 persons aged less than 20 years [22]. Three studies in Scandinavia showed a prevalence of 4.18 per 100,000 persons aged 18 years or less, and 3.23 and 2.78 per 100,000 persons aged below 16 years [23–25]. This could indicate regional differences in the incidence of SMA, i.e. gene pools. However, there are several other factors that may account for this observation. First of all, all studies were performed in small regions and thereby small populations were studied. For rare diseases like SMA, a small error in the detection of the number of cases can have a large impact on the estimated prevalence (sample bias). Secondly, these studies only took children into account, which is likely to influence the numbers in an upward direction. Furthermore, in the case of Sweden higher prevalence rates have also been observed in studies into other neuromuscular disorders, which could be due to a greater awareness and a good health system in Sweden, making it easier to identify patients for such a study [26–28]. A study in Northeast Saudi Arabia also found a very high prevalence rate. Although the prevalence of SMA might be different in the Middle East when compared to Europe, in more than half of the cases parental consanguinity was observed, which could at least partially explain the high prevalence [29].

### Prevalence by SMA subtype

Although SMA type I is expected to account for more than half of all new SMA cases [30], the studies that examined a SMA type I only showed a prevalence of 0.04 to 0.28 per 100,000 [24, 25, 31–34], which is much lower than the 1–2 per 100,000 persons noted for all SMA. Due to its severity, patients with SMA type I have a short life expectancy. Therefore often no or only few patients are alive on the date of the study, which could account for this lower prevalence. Nowadays, a median life expectancy of around one year of age is estimated for type I patients [35–37], whereas in type II 75–93% of patients survive beyond 20 years of age [37–40] and life expectancy for type III is thought to be close to the normal population [20, 39].

The prevalence of both SMA type II and III together has been estimated around 1.5 per 100,000 [31, 32, 41–43]. Of three studies that investigated type II and type III separately, two found a higher prevalence of type III compared to type II [24, 32]. This may be explained by the longer life expectancy of type III patients compared to type II SMA patients.

### Incidence

Incidence is the number of new cases of disease in a particular time period. In the case of SMA, the genotype is present at birth; a more precise term therefore is birth prevalence. Since newborn screening is not widely performed the number of patients expressing the phenotype is used instead to estimate the incidence. An overview of the studies examining the incidence is given in Table 3.

**Table 3:** Overview incidence of SMA by subtype

Country	Timeframe	SMA			Type I			Quality and comparability				Reference
		No of patients	Population	per 100,000	No of patients	Population	per 100,000					
Iceland	1982–1996	9	65,584	13.7	4	65,584	6.1					Ludvigsson_1999 [112]
Sweden (Western)	1980–2006	45	531,746	8.5	19	531,746	3.6	Genetic diagnosis used Only included patients below 16 years of age				Arkblad_2009 [23]
Sweden (Western)	1979–1994	21	343,941	6.1	13	343,941	3.8	Only included patients below 16 years of age				Darin_2000 [24]
Finland	1971–1985	68	952,228	7.1	53	952,228	5.6					Ignatius_1989 [51]
Estonia	1994–2003	15	129,832	11.6	9	129,832	6.9	Genetic diagnosis used				Vaidla_2006 [34]
United Kingdom (South Wales)	1973–1989	-	-	-	-	-	4.4	No details provided				MacMillan_1991 [113]
United Kingdom (Northeast England)	1966–1971	-	-	-	9	231,370	3.9	Type I defined as onset of disease before 12 months of age				Pearn_1973 [52]
Germany (West-Thüringen)	1974–1987	-	-	-	33	336,663	9.8					Thieme_1993 [33]
Switzerland	1960–1969	-	-	-	62	1,100,000	5.6	Probably also contains some type II patients				Zellweger_1972 [55]
Poland (Warsaw)	1976–1985	22	214,217	10.3	11	214,217	5.1	Patients described as type Ib reclassified as type II according to the description				Spiegler_1990 [42]
Poland	1998–2005	304	2,963,783	10.3	209	2,963,783	7.1	Genetic diagnosis used				Jedrzejowska_2010 [73]
Slovakia		-	-	17.8	-	-	8.1					Kvasnicova_1994 [44]
Hungary	1973–1980	-	-	-	91	1,376,928	6.6					Czeizel_1989 [50]
Italy (Northeast)	1960–1983	67	859,891	7.8	35	859,891	4.1					Mostacciuolo_1992 [114]
Italy (Bologna)	1970–1989	17	150,978	11.3	8	150,978	5.3	Only included patients below 20 years of age				Merlini_1992 [22]
Libya (Benghazi)	1983–1986	18	75,000	24	6	75,000	8	In half of the cases parental consanguinity was observed Population number is an estimation				Radhakrishan_1988 [48]
Reunion Island	1969–1980	-	-	-	19	24,000	79	In 13 siblings of a European community Population number is an estimation				Pascalet-Guideon_1984 [115]
Israel	1970–1975	-	-	-	4	1600	250	In an Egyptian Karaite community Population number is an estimation				Fried_1977 [116]
Canada (Toronto)	1955–1965	37	617,520	6.0	-	-	-					Winsor_1971 [111]
USA (Ohio)		4	40,103	10.0	-	-	-	Genetic diagnosis used				Prior_2010 [101]
USA (North Dakota)	1980–1987	-	-	-	14	94,092	14.9					Burd_1991 [53]
Cuba	1996–2002	51	1,018,454	5.0	36	1,018,454	3.5	Partly used genetic diagnosis				Zaldivar_2002 [45]
Country	Timeframe	Type II			Type III			Type II + III			Quality and comparability	Reference
		No of patients	Population	per 100,000	No of patients	Population	per 100,000	No of patients	Population	per 100,000		
Iceland	1982–1996	2	65,584	3.0	3	65,584	4.6	5	65,584	7.6		Ludvigsson_1999 [112]
Sweden (Western)	1980–2006	11	531,746	2.1	15	531,746	2.8	26	531,746	4.9	Genetic diagnosis used Only included patients below 16 years of age	Arkblad_2009 [23]
Sweden (Western)	1979–1994	3	343,941	0.9^b^	5	343,941	1.5	8	343,941	2.3	Only included patients below 16 years of age	Darin_2000 [24]
Finland	1971–1985	-	-	-	-	-	-	15	952,228	1.6		Ignatius_1989 [51]
United Kingdom (Northeast England)	1960–1969	-	-	-	-	-	-	15^k^	365,166	4.1		Pearn_1978 [41]
Germany (West-Thüringen)	1965–1980	-	-	-	-	-	-	42	395,646	10.6	Type II/III patients defined as: survival above 4 years of age	Thieme_1994 [43]
Poland (Warsaw)	1976–1985	10	214,217	4.7	1	214,217	0.5	11	214,217	5.1	Type II defined as onset of disease before 12 months of age	Spiegler_1990 [42]
Poland	1998–2005	37	2,963,783	1.2	58	2,963,783	2.0	95	2,963,783	3.2	Genetic diagnosis used	Jedrzejowska_2010 [73]
Slovakia		-	-	3.7	-	-	5.9	-	-	9.6		Kvasnicova_1994 [44]
Italy (Northeast)	1960–1983	19	859,891	2.2	13	859,891	1.5	32	859,891	3.7		Mostacciuolo_1992 [114]
Italy (Bologna)	1970–1989	8	150,978	5.3^f^	1	150,978	0.7	9	150,978	6.0	Only included patients below 20 years of age	Merlini_1992 [22]
Libya (Benghazi)	1983–1986	-	-	-	-	-	-	12	75,000	16	In half of the cases parental consanguinity was observed Population number is an estimation	Radhakrishan_1988 [48]

Incidence per 100,000 live births. Studies have been grouped by geographical region. Studies are based on clinical diagnosis, unless otherwise indicated. Indicated if described in article that different classification criteria than those in Table 1 were used

When evaluating the incidence of all types of SMA combined, on average an incidence of around 8 per 100,000 live births is found (~1 in 12,000). Some studies show a somewhat lower or higher incidence. In a study in Iceland an incidence of 13.7 per 100,000 live births was found. This is a study on an island with a relatively small population, where it might be easier to identify all patients. A study in Slovakia found a high incidence of 17.8 per 100,000, but details of the number of patients or population size were unavailable, making it difficult to interpret these findings [44]. In a recent study in Cuba a lower incidence of 5.0 per 100,000 was seen [45]. Patients were detected via an obligatory governmental registry and approximately 70% of the patients were genetically confirmed. This study also examined the ethnicity of the SMA type I patients. The majority of these patients were White (30/36), 5/36 were of mixed race and 1/36 patient was Black. Although this could be partially explained by the racial composition of the Cuban population, still relatively more White people were affected. There are several reasons that could account for this. First, there is a difference in incidence between various ethnicities. There are also reports of lower SMA carrier frequencies among Hispanics [46, 47]. However, it could also be the case that there are differences in the access to health care between different ethnicities. In a small study among 75,000 persons in Libya, a high incidence (24 per 100,000 live births) was found, and this may partially be explained by a high degree of consanguinity [48].

### Incidence by subtype

In 1991, Alan Emery published a review estimating the incidence for SMA type I to be around 4–6 in 100,000 (1 in 12,500–1 in 16,667) live births [49], which was based on only three studies [50–52]. We identified 17 studies, which taken together indicated an SMA I incidence of approximately 6 per 100,000. In the USA (North Dakota) in a study that pre-dated genetic testing, high incidence was observed (14.9 per 100,000); however this study was performed in a very small population, and any error in the accuracy of case identification may be associated with the high incidence. All patients studied were Caucasian and no consanguinity was observed [53]. In a regional study in Germany, a higher incidence of 9.8 per 100,000 was found [33]. In Libya, a high incidence, as found for total SMA, was not observed among type I patients (8.0 per 100,000) [48]. This is again based on a small population and could be due to a lack of awareness of SMA at the time the study was conducted. Furthermore, SMA type I patients might have been missed due to their short life span. In two small communities a very high incidence was observed. On Reunion Island in a European community a founder effect (loss of genetic variation that occurs when a new population is established by a very small number of individuals, which could lead to a high incidence if in one of these founders a mutation was present) was clearly seen, leading to an incidence of 79 per 100,000. In an Egyptian Karaite community in Israel, where in more than half of the affected families consanguinity was observed, an incidence of as high as 250 per 100,000 live births was found.

For type II and III, a high incidence of both types combined was observed (10.6 per 100,000) in a German study in the same region as the previously mentioned type I study that partially covered the same time period [33, 43]. The healthcare system in Germany may partly explain these observations. Furthermore, there might be regional differences in SMA incidence. The authors suggest that SMA might be more prevalent in central and Eastern Europe than in Western Europe. For type II and type III SMA the highest occurrence was observed in Libya (16 per 100,000) [48].

A study not added in Table 3 is a study from Kurland et al. in Rochester, USA, studying the period 1945–1954. This study found only one SMA type I patient and the calculations used the total population size instead of the number of live births to calculate the incidence. Furthermore, this total population consisted of only 30,000 persons [54].

The epidemiologic burden of SMA is not equally divided over the subtypes. In 2004 Ogino et al. reviewed several studies and calculated incidence rates of 5.83 per 100,000 live births for SMA type I, 2.66 per 100,000 live births for type II and 1.20 per 100,000 live births for type III. This implied that SMA type I, II and III constituted 60%, 27% and 12% of all SMA cases, respectively [30]. This overview included the study of Radhakrishan et al. in Libya, in which for half of the families parental consanguinity was observed [48]. In our analysis, we calculated the percentages in two ways yielding nearly identical results. First by only taking studies into account in which all types of SMA were studied separately, as this makes a direct comparison possible; and, secondly by taking all studies presented into account. In both cases this resulted in incidence rates of around 5.5, 1.9 and 1.7 per 100,000 for type I, II and III, respectively. This yields a percentage of around 60% for the incidence of SMA type I; with the remaining 40% of the cases equally divided between type II and type III. This indicates that SMA I indeed makes up the largest proportion of the total SMA.

### Considerations for comparing studies

To date, there are few studies of the prevalence and/or incidence of SMA, with a small number of these being recent. Most of the studies have been carried out in Europe. Furthermore, four of the ten studies done outside of Europe were performed in the countries with high consanguinity or small communities, thereby they are not considered to be representative of the overall SMA prevalence and incidence. No worldwide studies have been published to date.

A number of limitations should be taken into account when estimating prevalence/ incidence of SMA and comparing the presented studies. Most studies have been performed before 1995 when the genetic cause for SMA, deletion of the *SMN1* gene, was identified [3], where after genetic diagnosis was implemented. Therefore, most studies rely on the less accurate clinical diagnosis of SMA. This increases the chance of misdiagnosis of diseases with clinical features similar to SMA. Another difficulty comparing studies is that the classification of SMA has slightly changed over the years and it is not always clear which classification system has been used. For example, in the studies of John Pearn in Northeast England patients were defined as SMA type I if they had an onset of symptoms before the age of 12 months, so this might also include some early diagnosed SMA type II patients [41, 52]. Chronic SMA was classified as patients living beyond 18 months old. However, in the study in West-Thüringen, Germany patients had to survive till at least four years of age to be classified as chronic SMA [43]. This is further exemplified by the study of Spiegler et al. in Warsaw, Poland. In this study type Ib patients are mentioned, and are defined as patients diagnosed at birth or in the first months of life and living up to 30 years, whereas type II SMA was described as having an onset at the age of one year onwards [42]. In the study of Zellweger et al. in Switzerland it is not clearly specified which definitions were used, but it is conceivable that some type II patients are included in the numbers of type I patients [55]. Currently, the classification of the main subtypes: I, II and III (and sometimes IV) as described in Table 1 is used.

Another factor that should be taken into account is that the studies have been performed in different time periods. The natural history of SMA has changed over the years as the standards of care and associated outcomes have largely improved in the recent years. For example for type I comparison of studies showed the mean age of death increased from 8.8–10 months in studies performed before 1995 to 10.4 months up to 4 years in studies performed after 2000 [35, 36]. This is partly due to the availability of assisted ventilation (non-invasive or through tracheostomy) and of tube feeding through a gastrostomy [36].

Lastly, most of the studies have been performed in small geographical areas, thereby including a relatively small study population. One or two patients more or less in a small patient population will have a strong effect on the calculated prevalence or incidence. All these factors make a comparison between the studies and the interpretation of the findings difficult.

In conclusion, few prevalence and incidence studies have been performed for SMA, of which most are based on clinical diagnosis and are performed in European countries or regions, using small study populations. In addition to prevalence and incidence studies, carrier frequencies can provided useful additional information about, for example, ethnic subpopulations.

## Carrier frequency in SMA

**Fig. 1:**
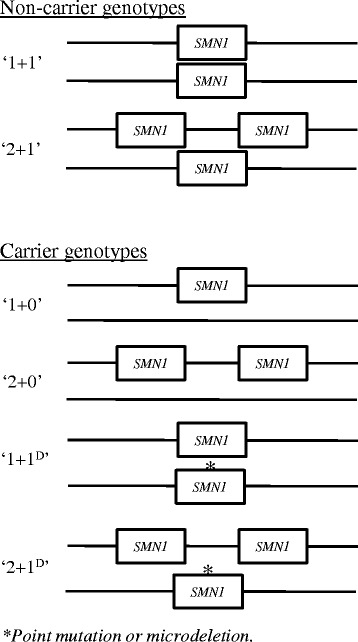
Most common SMA genotypes among non-carriers and carriers

Since SMA is a recessive disease, there are also unaffected, heterozygous carriers of the disease. Carriers fall into four main groups of genotypes (Fig. 1). The most common one is the ‘1 + 0’ genotype (one normal, functional allele and a *SMN1* deleted, disease allele). A much less common category is the ‘2 + 0’ genotype with two functional genes on one chromosome and none on the other. Furthermore, there are also ‘1 + 1^D^’ and ‘2 + 1^D^’ genotypes, which have one or two functional genes on one chromosome and a non-functional gene due to either a point mutation or a microdeletion on the other. These last two genotypes are very rare [56, 57]. Four or even more copies of the *SMN1* gene have also been found, indicating a ‘2 + 2’ or possibly a ‘3 + 1’ genotype. This suggests ‘3 + 0’ or ‘3 + 1^D^’ carrier genotypes might also be possible, however these will be even rarer.

No signs of disease have been associated with being a carrier for SMA. However, some studies suggest abnormal *SMN1* copy numbers (either deletions or duplications) may increase the risk and severity of sporadic amyotrophic lateral sclerosis (ALS), although other studies have been unable to confirm this association (for a review see Butchbach et al., 2016 [58]). Furthermore, it was suggested that in the rare disorder progressive muscular atrophy (PMA) *SMN1* duplications might be associated with a more severe clinical phenotype [59].

After the discovery of mutations in *SMN1* as the cause of SMA, several studies into the carrier status of SMA have been performed. In contrast to the prevalence/ incidence studies, most studies have been performed outside of Europe. Some of these are population screening programmes, whereas others are large samples of the general population [46, 60–81]. There are also studies where small population samples were analysed or the carrier frequency was estimated from healthy controls screened for *SMN1* for other purposes [8, 30, 82–99]. As mentioned before, frequencies estimated from a small population sample are less accurate. An overview of all studies is given in Additional file 2.

### Subpopulation differences

Some of the studies have examined differences between ethnic groups within their study population [46, 62–65, 77, 80]. The main finding was that copy numbers were significantly higher in Black (Sub-Saharan African ancestry) people. This was seen in African Americans [46, 62, 77], as well as in Black Africans [66] and would indicate a higher proportion of 2-copy (duplication) alleles, thereby suggesting a higher number of ‘2 + 0’ carriers. This could account for a lower detection rate (around 70% for Black people versus 90–95% for other ethnicities), leading to a high number of false negatives. The study in Africa found a significantly lower carrier frequency compared to Eurasians [66]. Lower carrier frequencies were also seen in a study comparing Black and White people in South Africa and a study among samples of the 1000 genome project [65, 80]. However, these studies could not detect the ‘2 + 0’ carriers, which could reduce the observed differences. Some studies also found lower carrier frequencies in Hispanics [46, 77], but this was not seen in other studies [62, 69, 80]. Lastly, Luo et al. identified a specific haplotype, present in Ashkenazi Jews and Asians detectable by microsatellite analysis, that could distinguish duplication alleles (present in ‘2 + 0’ carriers) from normal ‘1 + 1’ genotypes [77].

**Fig. 2:**
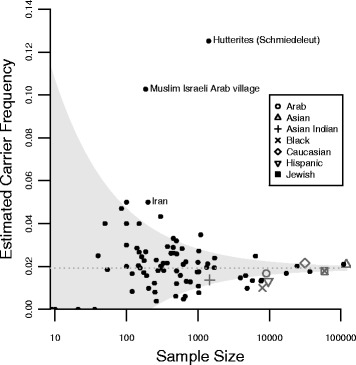
Carrier frequency studies for SMA. The *grey* area represents the 95% confidence interval based on the average carrier frequency (0.019) of all individuals (except those from the isolated Muslim Israeli Arab village and the Schmiedeleut Hutterites). *Small dots* represent individual studies. In case studies seperated between groups, these are depicted as separate dots. *Large symbols* represent pooled estimates for different ethnical groups

We carried out an analysis of differences between ethnic groups and studies. Fig. 2 shows a comparison of all studies described in Additional file 2 (ethnicities are indicated). The grey area indicates the 95% confidence interval based on the average carrier frequency of all studies combined (0.019).^1^ Most studies fall within this area, indicating no large differences in carrier frequency. Two populations (a Muslim Arab village in Israel and a specific group of Hutterites in South Dakota, USA) showed a particularly high carrier frequency. However, these are isolated populations with a high degree of inbreeding [81, 89]. Also in an Iranian population a higher carrier frequency was seen (1 in 20). However this is based on one study with a small sample size, furthermore, in Iran, consanguineous marriages are common [91]. Combined estimates of carrier frequencies for ethnic groups were calculated (large symbols in Fig. 2 and Table 4).

**Table 4:** Carrier frequencies for SMA per ethnicity

Population	Number of identified carriers	Number of study participants	Carrier frequency	95% CI
Arab^a^	152	9058	0.017	0.014–0.019
Asian	2492	119,718	0.021	0.020–0.022
Asian Indian	20	1465	0.014	0.008–0.020
Black (Sub-Saharan ancestry)	80	8012	0.010	0.008–0.012
Caucasian	680	31,549	0.022	0.020–0.023
Hispanic	127	9649	0.013	0.011–0.015
Jewish	1059	59,196	0.018	0.017–0.019

Median and 95% posterior intervals of SMA carrier frequencies, based on studies presented in Additional file 2. Only ethnicities studied in more than one study are included

*CI* confidence interval

^a)^ The isolated Muslim Israeli Arab village [59] has been omitted

The results show that the highest frequencies are found in Caucasian and Asian populations (around 1 in 50) and the lowest in Black (1 in 100) and Hispanics (1 in 76) populations. However, it is important to note that genetically Hispanics are a very mixed group, making generalizations difficult. This is also demonstrated by the fact that some studies among Hispanics found much higher frequencies [69, 80], while others found that the frequencies were lower [46].

### *SMN1* copy number differences between populations

In 2014, MacDonald et al. have performed a meta-analysis comparing the SMA carrier frequency among different ethnicities. In their analysis they included 14 studies where ethnicities were described and results were broken down by SMA copy number [47]. They took the different carrier genotypes described above into account and determined the carrier rates in the ethnic groups. Furthermore, they calculated the reduced risk of being a carrier if a 2- or 3-copy result was found. This again showed a substantially higher carrier risk with a 2-copy test result for Black people. In addition a very high carrier risk and 2-copy risk in Iranians was found. However, this is based on one study only [91].

The Additional file 3 shows all studies that examined at the *SMN1* copy number status. None of the studies among Arabian populations performed this analysis, therefore this group has not been included in the table. *SMN1* allele frequencies were determined for each group (Table 5) using copy numbers (for methods and calculations see Additional file 1).

**Table 5:** *SMN1* allele frequencies per ethnicity

	Frequency by number of *SMN1* copies on a single allele					
	0 Copies		1 Copy		2 Copies	
Ethnicity^a^	Portion	95% CI	Portion	95% CI	Portion	95% CI
Asian	0.010	0.010–0.011	0.949	0.948–0.950	0.040	0.039–0.041
Asian Indian^b^	0.010	0.006–0.015	0.908	0.894–0.921	0.082	0.070–0.095
Black (Sub-Saharan ancestry)	0.007	0.005–0.009	0.717	0.708–0.725	0.276	0.268–0.285
Caucasian	0.011	0.010–0.012	0.954	0.952–0.955	0.035	0.034–0.037
Hispanic	0.007	0.006–0.008	0.915	0.911–0.919	0.078	0.074–0.082
Jewish	0.010	0.009–0.011	0.934	0.931–0.936	0.057	0.054–0.059

Median and 95% posterior intervals of copy number allele frequency, based on studies presented in Additional file 3

*CI* confidence interval

^a)^ Same ethnicities as in Table 4 are included

^b)^ Only consists of one study

The copy number 0 (carriers) is lower in Blacks and Hispanics. Whilst there is not a great difference in the two copy number frequencies between other ethnicities, this is much higher in the Black population. As is seen in Table 6, this indicates a higher number of hidden carriers (‘2 + 0’ genotype), thereby decreasing the sensitivity of most carrier tests used, which only measure the copy numbers. Therefore, it is important to take the ethnicity into account when performing population screening or genetic counselling and consider a different method to reduce the chance of false negative results. In Table 6 also disease frequencies are estimated by combining the copy number results with an estimated small mutation (1^D^) frequency of 4% [4, 5] and an estimated de novo mutation frequency of 2% [100]. Thereafter, the incidences rates were estimated using these frequencies (Table 7).

**Table 6:** Carrier, SMN1 copy number 2 carrier and disease frequencies per ethnicity

	Adjusted carrier and disease frequencies					
	1 *SMN1* copy carriers		2 S*MN1* copies carriers		Disease Frequency	
Ethnicity	Frequency	95% CI	Frequency	95% CI	Frequency	95% CI
Asian	0.022	0.021–0.023	0.0014	0.0012–0.0015	1.3E-04	1.2E-04-1.4E-04
Asian Indian^a^	0.020	0.012–0.030	0.0021	0.0014–0.0030	1.0E-04	4.0E-05-2.4E-04
Black (Sub-Saharan ancestry)	0.014	0.011–0.018	0.0042	0.0034–0.0052	5.3E-05	3.4E-05-8.1E-05
Caucasian	0.022	0.021–0.024	0.0013	0.0011–0.0014	1.3E-04	1.1E-04-1.5E-04
Hispanic	0.014	0.011–0.017	0.0015	0.0013–0.0018	5.0E-05	3.5E-05-7.0E-05
Jewish	0.020	0.018–0.022	0.0016	0.0014–0.0018	1.0E-04	8.3E-05-1.2E-04

Median and 95% posterior intervals of carrier, copy number 2 carriers and disease frequencies. Frequencies calculated based on allele frequencies, small mutation and de novo mutation frequencies (calculations described in Additional File 1)

*CI* confidence interval

^a)^ Based on results of only one study

**Table 7:** Estimated incidence from carrier frequency per ethnicity

Ethnicity	Incidence estimate (1 in)	95% CI
Asian	8009	7382–8684
Asian Indian^a^	9655	4227–25,057
Black (Sub-Saharan ancestry)	18,808	12,331–29,559
Caucasian	7829	6750–9093
Hispanic	20,134	14,218–28,894
Jewish	10,000	8343–12,038

Median and 95% posterior intervals of incidence estimated from carrier and disease frequencies as calculated in Table 6

a) Based on results of only one study

The incorporation of estimated carrier risks for people with a 2 copy number result, generates only a slightly lower incidence (~1 in 54) for the Black populations compared to most other populations (~1 in 45), due to presence of a much higher number of multiple *SMN1* copy number alleles in this population. The estimation of the combined carrier frequency in Hispanics is lower than in other populations (1 in 65), as was also seen in the previous estimations. It must be noted however that here only a subset of studies is used compared to the comparison of all studies (Fig. 2 and Table 4), which can also contribute to differences in estimations.

The combined results lead to the highest incidence estimations of around 1 in 8000 in Asians and Caucasians, whilst lower incidence of around 1 in 20,000 are estimated in the Black and Hispanic populations.

In Caucasians, the incidence rate estimated from carrier frequencies is higher than the observed incidence rates in studies (Table 3, ~1 in 11,000). Carrier frequency estimates are solely based on genetic studies, whilst most incidence studies were based on clinical diagnosis and are mostly much older. However, carrier frequency incidence estimates could be an overestimation of the true incidence due to reduced penetrance. Here a penetrance of 100% is assumed. If the penetrance is decreased by 10% (i.e. penetrance of 90%) the incidence would also decrease by 10%. It might be that some cases of SMA are so severe that they lead to premature death in utero. SMN2 is absent in 10–15% of the general population [101], and deletions of both *SMN1* and *SMN2* are embryonically lethal. Furthermore increased awareness could lead to more genetic counselling of couples at risk, certainly in couples who have previous children or family members with SMA. In addition, sporadic cases of unaffected individuals without functional *SMN1* cases have been described [96, 102–109]. This might be due to high copy numbers of *SMN2*, since, as mentioned before, *SMN2* copy number influences the severity of the disease [7–9]. Therefore, it is important to take *SMN2* copy number into account when performing newborn screening.

## Conclusions

SMA is a severe, heterogeneous, neuromuscular disorder. The few available prevalence and incidence studies mainly predate genetic testing and were performed in small geographical areas, mainly in Europe. This highlights the need for larger, more generalizable prevalence studies.

Recently, carrier frequency of SMA in healthy populations has been studied quite extensively, indicating differences between ethnicities not only in carrier frequency, but also in copy number status. In some groups this decreases the sensitivity of commonly used carrier testing methods. This emphasizes the need to use methods that enable to detect carriers having two *SMN1* copies on one chromosome and none on the other.

Good epidemiological data is needed to gain insight into health care needs and for research studies and clinical trials. This is especially important in rare diseases where clinical trials require a careful planning. Furthermore, newborn screening will become increasingly important, especially now a drug has been approved and other new therapies are in advanced clinical trial stages. The introduction of new therapies is also likely to impact on the prevalence of SMA and as such may have significant resource implications for health care planning.

## Additional files

Additional file 1: Bayesian analysis of copy number frequency, small mutations and de novo mutation rate. (DOCX 26 kb)

Additional file 2: Table S1. Overview carrier frequencies of SMA. (XLS 94 kb)

Additional file 3: Table S2. Copy numbers SMN1 gene in general population. (XLS 52 kb)

## Abbreviations

ALS: Amyotrophic lateral sclerosis
PMA: Progressive muscular atrophy
SMA: Spinal muscular atrophy
SMN: Survival motor neuron

## Footnotes

1 Two studies in studies in populations with a high inbreed rate (a Muslim Arab village in Israel and a Hutterite community in South Dakota, USA) are not included in the average.
