# Supplementary material for: Prevalence, incidence and carrier frequency of 5q–linked spinal muscular atrophy – a literature review
Source: Orphanet J Rare Dis. 2017 Jul 4;12:124. doi: 10.1186/s13023-017-0671-8 (PMC5496354; doi:10.1186/s13023-017-0671-8)
Supplement: Supplementary file 1 — Bayesian analysis of copy number frequency, small mutations and de novo mutation rate. (DOCX 26 kb) [file 13023_2017_671_MOESM1_ESM.docx]

**Additional file 1:** Bayesian analysis of copy number frequency, small mutations and de novo mutation rate

**Statistical modelling**

The model consist of different parts: genotype copy number frequencies, copy number 1 disease alleles containing small mutations (point mutations or microdeletions) and de novo mutation frequency.

**Copy number frequency**

Since not all studies differentiate between copy number 3 and 4, we pool copy numbers 3 and 4. Simulation studies (data not shown) have shown that this makes little difference to point estimates of frequency and increases the variability slightly. Hardy-Weinberg equilibrium is assumed. Assuming copy numbers 0, 1 and 2 per allele are present differing between ethnicities, the frequency of copy number *j* in population *I* is:

$$p_{i0}+p_{i1}+p_{i2}=1$$

**Small mutations**

For estimating the small mutation frequency data from Wirth *et al.* and Alías *et al.* is used [1, 2]. The relative frequency of individuals with small mutations ($\beta$) is estimated from the copy number 1 alleles with a small mutation ($p_{1}^{D}$):

$$\beta=2p_{1}^{D}/(p_{0}+p_{1}^{D})$$

*Z_i_* is the number of individuals with a small mutation out of $M_{i}$ for study $i$.

**De novo mutations**

To estimate the de novo mutation rate used in our analysis, the observed mutation rate in Wirth *et al.* is used [3], accounting for uncertainty. The mutation rate ($\mu$) is estimated using the disease rate for individuals with non-carrier parents ($r$) and the frequency of the disease allele ($q$):

$$\mu=\frac{rq}{(2-2r)}$$

Taking a disease allele frequency of $q=0.001$, gives an estimation of $\pi\approx0.0001$. This approximate mutation rate is supported by population genetic theory. If the SMA deletion mutation had reached mutation-selection balance, the mutation rate *μ* would be approximately equal to $q^{2}$ [4]. $q^{2}={0.01}^{2}\approx0.0001$, which is in the same order of magnitude.

**Bayesian model**

Ignoring ethnicity, in $k$ studies for which $X_{ij}$ is the number of individuals in study $i$ with $j$ copies and $N_{i}=\sum^{j} X_{ij}$. Assuming Hardy-Weinberg equilibrium, we write the (unobserved) genotype probabilities for 1, 2, and 3+ copies as $y_{1}$, $y_{2}$, and $y_{3+}$, and the genotype frequencies are multinomially distributed. A uniform Dirichlet prior for the copy number frequencies per allele is used.

The data and models above, lead to the following set of equations:

$$y_{i1}=2p_{i0}p_{i1}$$

$$y_{i2}=p_{i_{1}}^{2}+2p_{i0}p_{i2}$$

$$y_{i3+}=2_{pi2}p_{i1}+p_{i2}^{2}$$

$$X_{i}\sim\mathrm{Multinomial}(N_{i},\beta)$$

$$\beta=\frac{2p_{1}^{D}}{p_{0}+2p_{1}^{D}}$$

$$Z_{i}\sim\mathrm{Binomial}\left( M_{i},\beta\right)$$

$$r=\frac{2\pi\left( 1-p_{0} \right)}{p_{0}+2\mu\left( 1-p_{0} \right)}$$

$$Y\sim\mathrm{Binomial}\left( S,r \right)$$

**Inference**

Due to the complexity of this model likelihood calculations are not possible. Markov chain Monte Carlo using the JAGS package within R version 3.32 [5] is used to draw samples is used to draw samples from the posterior distribution of the parameters of interest [6]. Details and code of the model are available at <http://rpubs.com/ijwilson/242602>.

A Bayesian approach to the problem with symmetric Dirichlet priors on all allele frequencies with parameter 1, an exponential prior on the mutation rate with a mean of 0.0001, and a beta prior with mean 0.01 on the proportion of copy number 1 alleles carrying a recessive SMA causing small mutation is used. These priors are quite diffuse and different priors make little difference to our inference.

$$p\sim\mathrm{Dirichlet}\left( 1,1,1 \right)$$

$$p_{1}^{D}\sim\mathrm{Beta}(1,100)$$

$$\mu\sim\mathrm{Gamma}(1,1000)$$

This Bayesian analysis gives posterior samples from all parameters, which are then summarised to obtain estimates of our quantities of interest, with the corresponding levels of uncertainty in our estimates. For point estimates 95% posterior intervals are used.

These estimates can be straightforward, such as the frequency of the copy number 0 allele between different populations, or can be more involved, such as the example below.

**Example: Probability of disease**

By combining all the inferences together we can get estimates of quantities that we are interested in, from these analyses that include the uncertainty from our estimates of the copy number frequency, the small mutation frequency and the de novo mutation rate.

For example we can ask: “What is the probability that a child at random from a population is affected?” Such an estimate depends on knowledge about the frequency of copy number variants, the frequency of small mutations and the rate of de novo mutations.

$$P\left( \mathrm{disease} \right)=p_{carrier}^{2}\left( 0.25+0.5\mu+0.25\mu^{2} \right)+2p_{carrier}\left( 1-p_{carrier} \right)\left( 0.5\mu+0.5\mu^{2} \right)+\mu^{2}{(1-p_{carrier})}^{2}$$

where $p_{carrier}=2\left( p_{0}+p_{1}^{D} \right)1-p_{0}-p_{1}^{D})$.

**References**

1. Wirth B, Herz M, Wetter A, Moskau S, Hahnen E, et al. Quantitative analysis of survival motor neuron copies: identification of subtle SMN1 mutations in patients with spinal muscular atrophy, genotype-phenotype correlation, and implications for genetic counseling. Am J Hum Genet. 1999; 64:1340-56.

2. Alias L, Bernal S, Fuentes-Prior P, Barcelo MJ, Also E, et al. Mutation update of spinal muscular atrophy in Spain: molecular characterization of 745 unrelated patients and identification of four novel mutations in the SMN1 gene. Hum Genet. 2009; 125:29-39.

3. Wirth B, Schmidt T, Hahnen E, Rudnik-Schoneborn S, Krawczak M, et al. De novo rearrangements found in 2% of index patients with spinal muscular atrophy: mutational mechanisms, parental origin, mutation rate, and implications for genetic counseling. Am J Hum Genet. 1997; 61:1102-11.

4. Crow JF, Kimura M: An introduction to population genetics theory. New York: Harper & Row; 1970.

5. R-CoreTeam. R: A Language and Environment for Statistical Computing, <https://www.R-project.org>. Vienna, Austria: R Foundation for Statistical Computing; 2016.

6. Plummer M. JAGS: A program for analysis of Bayesian graphical models using Gibbs sampling. Proceedings of the 3rd international workshop on distributed statistical computing. 2003; 124.
